# Inflammatory monocytes damage the hippocampus during acute picornavirus infection of the brain

**DOI:** 10.1186/1742-2094-9-50

**Published:** 2012-03-09

**Authors:** Charles L Howe, Reghann G LaFrance-Corey, Rhianna S Sundsbak, Stephanie J LaFrance

**Affiliations:** 1 Department of Neurology, Mayo Clinic College of Medicine, Rochester, MN 55905, USA; 2 Guggenheim 15-01A, Mayo Clinic, 200 First St SW, Rochester, MN 55905, USA

**Keywords:** 1A8, alveus, brain-infiltrating leukocytes, CD11b, Gr1, hippocampus, inflammatory monocyte, macrophage, neutrophil, LysM:GFP reporter mouse, Ly6C, Ly6G, Theiler's virus

## Abstract

**Background:**

Neuropathology caused by acute viral infection of the brain is associated with the development of persistent neurological deficits. Identification of the immune effectors responsible for injuring the brain during acute infection is necessary for the development of therapeutic strategies that reduce neuropathology but maintain immune control of the virus.

**Methods:**

The identity of brain-infiltrating leukocytes was determined using microscopy and flow cytometry at several acute time points following intracranial infection of mice with the Theiler's murine encephalomyelitis virus. Behavioral consequences of immune cell depletion were assessed by Morris water maze.

**Results:**

Inflammatory monocytes, defined as CD45^hi^CD11b^++^F4/80^+^Gr1^+^1A8^-^, and neutrophils, defined as CD45^hi^CD11b^+++^F4/80^-^Gr1^+^1A8^+^, were found in the brain at 12 h after infection. Flow cytometry of brain-infiltrating leukocytes collected from LysM: GFP reporter mice confirmed the identification of neutrophils and inflammatory monocytes in the brain. Microscopy of sections from infected LysM:GFP mice showed that infiltrating cells were concentrated in the hippocampal formation. Immunostaining confirmed that neutrophils and inflammatory monocytes were localized to the hippocampal formation at 12 h after infection. Immunodepletion of inflammatory monocytes and neutrophils but not of neutrophils only resulted in preservation of hippocampal neurons. Immunodepletion of inflammatory monocytes also preserved cognitive function as assessed by the Morris water maze.

**Conclusions:**

Neutrophils and inflammatory monocytes rapidly and robustly responded to Theiler's virus infection by infiltrating the brain. Inflammatory monocytes preceded neutrophils, but both cell types were present in the hippocampal formation at a timepoint that is consistent with a role in triggering hippocampal pathology. Depletion of inflammatory monocytes and neutrophils with the Gr1 antibody resulted in hippocampal neuroprotection and preservation of cognitive function. Specific depletion of neutrophils with the 1A8 antibody failed to preserve neurons, suggesting that inflammatory monocytes are the key effectors of brain injury during acute picornavirus infection of the brain. These effector cells may be important therapeutic targets for immunomodulatory or immunosuppressive therapies aimed at reducing or preventing central nervous system pathology associated with acute viral infection.

## Background

Viral infection of the central nervous system (CNS) may induce clinically relevant outcomes that range from coma, paralysis, and death to persistent cognitive impairment, seizures, and epilepsy [1]. Many viral infections of the CNS are acute, with viral clearance mediated by the adaptive arm of the immune system. However, the relationship between the delayed adaptive antigen-specific T and B cell-mediated response that eventually controls and eliminates the viral pathogen and the rapid but largely non-specific innate immune system response is poorly understood. Indeed, different viruses and different hosts exhibit disparate relationships between the innate and adaptive response to infection. For example, McGavern and colleagues have shown in mice infected with lymphocytic choriomeningitis virus that pathogenic neutrophils and inflammatory monocytes are recruited to the brain by antiviral CD8^+ ^T cells. Depletion of the CD8^+ ^T cell response reduced the neutrophil and inflammatory monocyte burden in the CNS and delayed pathogenesis [2]. In contrast, Bergmann and colleagues observed that neutrophils and inflammatory monocytes were the first leukocytes to infiltrate the brain in mice infected with the non-lethal neurotropic JHM strain of mouse hepatitis virus (JHMV) [3], and depletion or blockade of monocytes impaired subsequent T cell infiltration into the brain parenchyma [4]. Lane and colleagues also observed a very early neutrophil response to CNS infection with JHMV, and in contrast to the Bergmann *et al. *findings implicating monocytes, they found that blocking neutrophil entry into the CNS resulted in impairment of the subsequent T cell response [5]. Finally, Lokensgard and colleagues observed an early neutrophil and inflammatory monocyte response in the brain following infection with herpes simplex virus 1 and this response preceded lymphocyte infiltration by a week [6]. Overall, the common denominator in all of these studies was the ensuing neuropathology triggered by infiltrating neutrophils and inflammatory monocytes.

Our previous experience with the Theiler's murine encephalomyelitis virus (TMEV) model of acute picornavirus infection of the CNS [7] indicated that brain pathology and the functional sequelae of such injury occur as a result of an early, preadaptive immune response. Indeed, hippocampal pyramidal neurons were clearly injured at 1 day after infection [8]. We have hypothesized that neutrophils and inflammatory monocytes, as part of an early wave of first responders to infection, are responsible for hippocampal injury and loss of memory function observed in TMEV-infected mice, but the relative contribution of each population to this injury was unclear. In the present study we phenotyped the brain-infiltrating leukocyte population at several acute time points after infection with TMEV. We found that neutrophils and inflammatory monocytes were present in the brain within 12 h of infection, indicating that in this model system infiltration of such innate effectors is a hyperacute response. Furthermore, we found that the absence of an inflammatory monocyte response but not the absence of a neutrophil response resulted in neuroprotection and cognitive preservation.

## Methods

### Virus

At 5 to 8 weeks of age, mice were infected by intracranial injection of 2 × 10^5 ^plaque-forming units (PFU) of the Daniel's strain of the Theiler's murine encephalomyelitis virus (TMEV) in 10 μL RPMI (the media used to grow the virus) [9]. When relevant, sham-infected mice received intracranial injection of 10 μL virus-free RPMI.

### Mice

C57BL/6/J (no. 000664) mice aged 4 to 6 weeks were acquired from The Jackson Laboratories (Bar Harbor, ME, USA). Upon arrival, mice were acclimatized for at least 1 week prior to use. Breeding pairs of LysM:eGFP mice [10] were kindly provided by Dr. David Sacks (National Institutes of Health/National Institute of Allergy and Infectious Diseases) and bred in house. Mice were group housed in the Mayo Clinic College of Medicine research vivarium under conventional conditions with ad libitum access to food and water. Sex was mixed for all experiments. All animal experiments conformed to the National Institutes of Health and Mayo Clinic Institutional Animal Care and Use Committee guidelines.

### Histology and immunostaining

Following intraperitoneal injection of a terminal dose of pentobarbital (100 mg/kg), mice were perfused via intracardiac puncture with 50 mL of 4% paraformaldehyde in phosphate-buffered saline (PBS). For paraffin sections, the brain was postfixed in 4% paraformaldehyde at 4°C for 24 h and then blocked via coronal cuts at the level of the optic chiasm and infundibulum. Tissue blocks were embedded in paraffin, sectioned at 5 microns, mounted on charged slides, rehydrated, and stained with hematoxylin and eosin. For vibratome sections, the brain was postfixed in 4% paraformaldehyde at 4°C for 6 h and then blocked to isolate the hippocampal field. Tissue blocks were embedded in agar and sectioned at 80 microns. Free floating sections were blocked in PBS plus 10% normal donkey serum for 1 h, incubated overnight at 4°C with primary antibodies diluted 1:100 in block, washed, incubated with fluorescently tagged secondary antibody diluted 1:200 in PBS, washed, and mounted on gelatin-subbed slides. CD45 was detected with clone 30-F11 (BD Biosciences, San Jose, CA, USA). CD11b was detected with clone M1/70 (BD Biosciences). Ly6C/G was detected with clone Gr1, RB6-8C5 (BD Biosciences). Ly6G was detected with clone 1A8 (BD Biosciences).

### Brain-infiltrating leukocyte preparation

Our published protocol was followed with slight modification [9]. Briefly, homogenized brain material was centrifuged through a 30% Percoll gradient at 7,800 *g*_ave _for 30 minutes without braking. The washed and strained cell suspension was then centrifuged on a 1.100 g/mL Percoll layer for 20 minutes at 800 *g*_ave_. The interface containing neutrophils and inflammatory monocytes was collected, washed, and used for flow cytometry.

### Flow cytometry

Flow cytometry buffer contained 1% bovine serum albumin and 0.02% sodium azide in PBS. Blocking buffer contained flow cytometry buffer, supernatant from the 2.4G2 hybridoma (Fc block; anti-CD16/32; American Type Culture Collection, Manassas, VA, USA no. HB-197), and fetal bovine serum at a ratio of 10:5:1. After isolation, cells were blocked at 4°C for 30 minutes. Primary antibodies were used at 1:200 and incubated for 30 minutes at 4°C. Stained cells were washed three times in flow cytometry buffer and fixed in 2% paraformaldehyde prior to flow cytometric analysis on a BD FACSCalibur (BD Biosciences). Files were analyzed offline using FlowJo 7.5 (Windows version; Tree Star, Inc., Ashland, OR, USA). CD45 was detected with clone 30-F11 (BD Biosciences no. 557235). CD11b was detected with clone M1/70 (BD Biosciences no. 553312). F4/80 was detected with clone BM8 (eBiosciences no. 53-4801-82). Ly6C/G was detected with clone Gr1, RB6-8C5 (BD Biosciences no. 553129). Ly6G was detected with clone 1A8 (BD Biosciences No. 551467). Ly6B was detected with clone 7/4 (Caltag no. RM6504). Ly6C was detected with clone AL-21 (BD Biosciences no. 560595).

### Morris water maze

Cognitive performance was assessed beginning at 14 days postinfection using our previously published methodology [1].

### Data analysis

All graphs show mean ± 95% confidence intervals. Cell counts in the depletion experiments were assessed by one-way analysis of variance (ANOVA). Morris water maze performance was assessed by two-way ANOVA. Pairwise analyses, when appropriate, used the Student-Newman-Keuls method. All tests utilized α = 0.05, β = 0.2.

## Results and discussion

### Immune cells rapidly infiltrate the brain of TMEV-infected mice but not sham-infected mice

**Figure 1:**
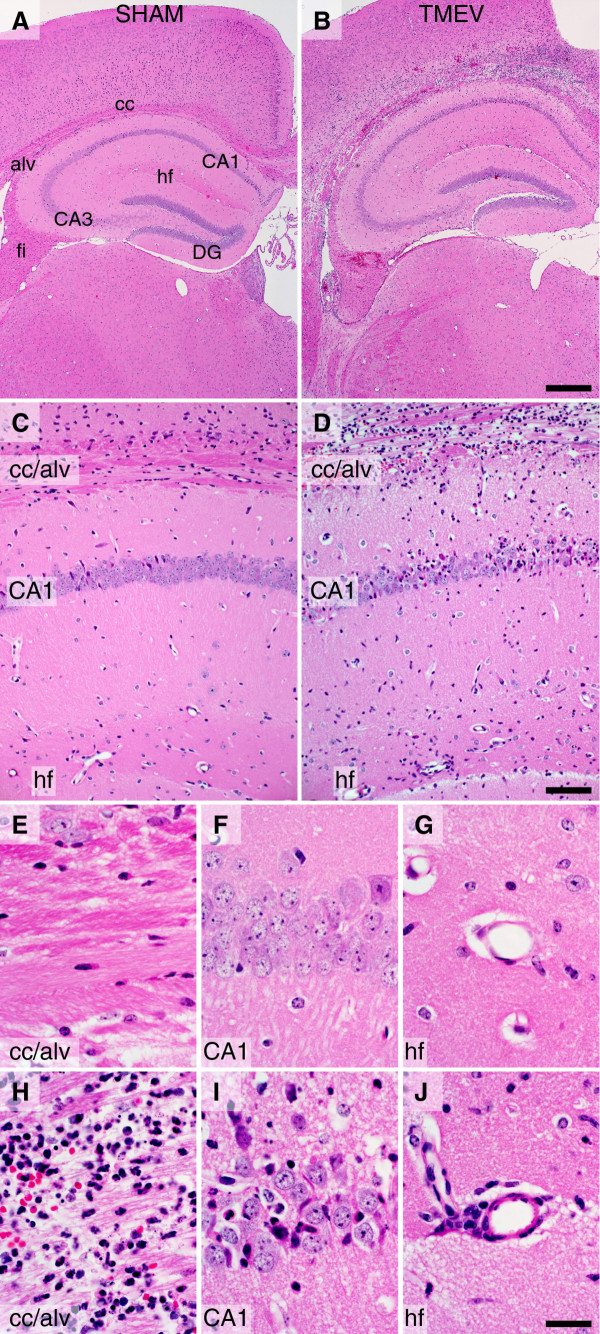
**Histological evidence of rapid immune cell infiltration into the hippocampus of mice acutely infected with Theiler's murine encephalomyelitis virus (TMEV)**. C57BL/6J mice were injected intracerebrally with 2 × 10^5 ^PFU TMEV in 10 μL of RPMI (B, D, H-J) or with 10 μL RPMI (SHAM) (A, C, E-G). The animals were killed by intracardiac perfusion of 4% paraformaldehyde 24 h after infection. Brain was processed for paraffin embedding and sectioning, and stained with hematoxylin and eosin. Robust inflammatory infiltrate is present above the hippocampus and throughout the corpus callosum in the TMEV-infected mice (B, D). Sham-infected mice exhibit no signs of infiltrate (A, C). Higher magnification confirms the presence of infiltrate throughout the corpus callosum and alveus (H), within the CA1 pyramidal neuron cell layer (I), and within the perivascular space along the hippocampal fissure (J) in virus-infected mice but not in sham-infected mice (E-G). Scale bar in B is 500 μm and refers to (A); scale bar in (D) is 100 μm and refers to (C); scale bar in (J) is 20 μm and refers to (E-I). Abbreviations: alv = alveus; CA1 and CA3 = cornu ammonis fields of the hippocampus; cc = corpus callosum; DG = dentate gyrus; fi = fimbria; hf = hippocampal fissure. Findings are representative of more than 20 animals per group.

C57BL/6J mice at 5 to 8 weeks of age were infected by intracranial injection of 2 × 10^5 ^PFU of the Daniel's strain of TMEV in a volume of 10 μL. Sham-infected mice received identical injections of vehicle lacking virus. Histological analysis of the brain at 24 h post infection (hpi) revealed marked inflammation and large numbers of infiltrating cells in the infected mice (Figure 1). Inflammation was most concentrated in the hippocampal region (Figure 1B), with high density in the corpus callosum and alveus and at the hippocampal fissure (Figure 1B, D). Infiltrating cells were also observed within the pyramidal neuron layer of cornu ammonis 1 (CA1) in the hippocampus (Figure 1D, I). In sham-infected mice, despite receiving a needle stick through the calvarium, no evidence of inflammation was ever observed in the brain. We also never observed an inflammatory response in mice injected with UV-inactivated TMEV (data not shown). We conclude that productive infection of the brain with TMEV results in the rapid induction of immune cell infiltration that is concentrated in the white matter above the hippocampus and at the hippocampal fissure.

### Brain infiltrating cells are enriched in neutrophils and inflammatory monocytes

**Figure 2:**
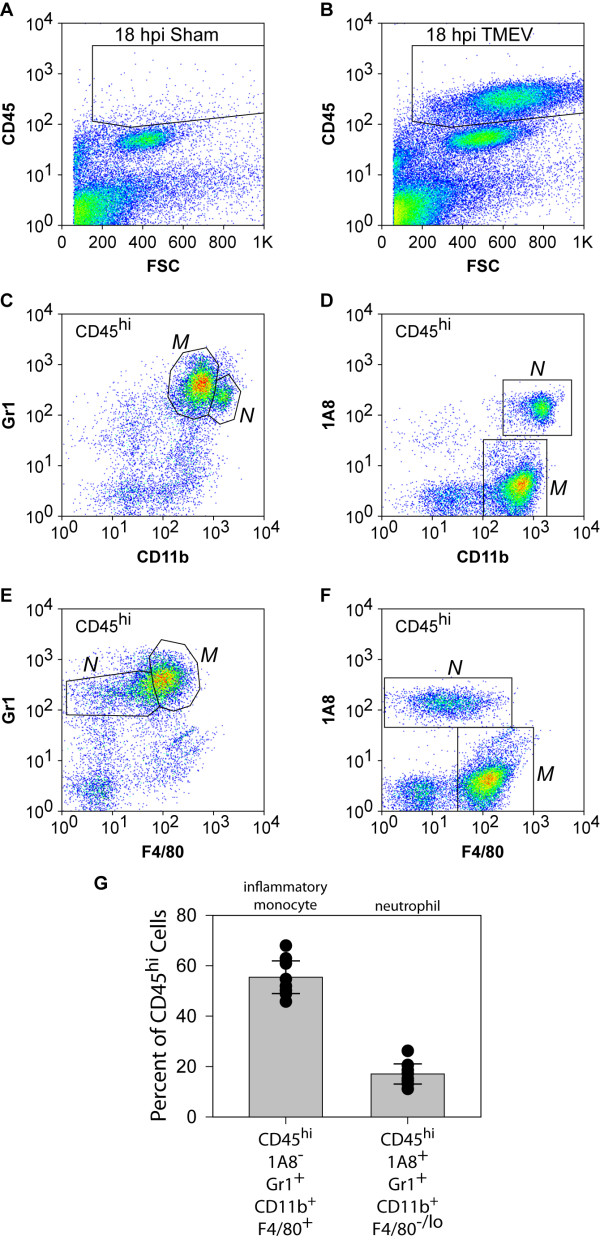
**Flow cytometric assessment of the infiltrate present in the brain of mice acutely infected with Theiler's murine encephalomyelitis virus (TMEV)**. TMEV-infected (B-G) or sham-infected (A) mice were killed at 18 h post infection (hpi). Brain was collected fresh and processed for the isolation of brain-infiltrating leukocytes (BILs). BILs were stained with antibodies against CD45, CD11b, F4/80, Gr1 (Ly6C/G) and 1A8 (Ly6G) and analyzed by flow cytometry. No CD45^hi ^cells were observed in sham-infected mice (A). In contrast, TMEV-infected mice exhibited a large population of CD45^hi ^cells at 18 hpi. Analysis of the CD45^hi ^cells in BILs from infected mice revealed the presence of CD11b^++ ^cells that were also strongly positive for the Gr1 antigen (C) and CD11b^+++ ^cells that were positive for both the Gr1 antigen (C) and the 1A8 antigen (D). In addition, a population of the Gr1^+ ^cells was also positive for F4/80 (E); no 1A8^+ ^cells were F4/80^+ ^(F). Combined gating revealed that CD45^hi^CD11b^++^Gr1^+ ^cells were also F4/80^+ ^while CD45^hi^CD11b^+++^Gr1^+ ^cells were F4/80^-^. Quantitation of neutrophils (CD45^hi^1A8^+^Gr1^+^CD11b^+++^F4/80^-^) and inflammatory monocytes (CD45^hi^1A8^-^Gr1^+^CD11b^++^F4/80^+^) in BILs from 10 individual mice revealed that about 80% of the CD45^hi ^cells belonged to one of these populations (G). Error bars in (G) represent 95% confidence intervals; black circles represent individual animals; 10 mice per group were analyzed and the flow plots in (A-F) are representative.

Given the conflicting observations that exist in the literature regarding temporal staging of innate and adaptive immune cell infiltration into the acutely infected brain [2-5,11], we sought to determine the identity of the cells observed in Figure 1. Using our established protocol for isolating a highly enriched population of leukocytes from the brain [9], we observed a robust population of CD45^hi ^cells in TMEV-infected mice at 18 hpi (Figure 2B) that are not present in the brain of sham-infected (Figure 2A) or uninfected mice (data not shown). Further phenotyping revealed that most of the CD45^hi ^cells in the brain-infiltrating leukocytes (BILs) were CD11b positive, albeit at two different intensity levels. We routinely distinguished CD11b^++ ^and CD11b^+++ ^populations (Figure 2C, D). Critically, these populations could also be distinguished by levels of surface staining with the Gr1 antibody (Figure 2C) and the 1A8 antibody (Figure 2D), such that CD11b^++ ^cells were positive for Gr1 but negative for 1A8 and CD11b^+++ ^cells were positive for both markers (Figure 2C, D). Likewise, the population of CD45^hi ^cells could be distinguished by surface F4/80 expression, with some F4/80^+ ^cells positive for Gr1 but no F4/80^+ ^cells positive for 1A8. CD11b is a component of the α_M_β_2 _integrin complex that is found on the surface of monocytes, granulocytes, macrophages, and natural killer cells [12]. The F4/80 antigen is a member of the epidermal growth factor 7 transmembrane family that is found on various macrophage populations including tissue resident macrophages such as microglia [13,14]. The Gr1 antigen is a mixture of Ly6C and Ly6G that is expressed on the surface of circulating monocytes and neutrophils [15]. In contrast, the 1A8 antigen is explicitly Ly6G and is only observed on neutrophils and granulocytes but not on monocytes or macrophages [15,16]. On the basis of our findings, we have established the following definitions, which are also elaborated below: CD45^hi^CD11b^++^F4/80^+^Gr1^+^1A8^- ^cells are inflammatory monocytes; CD45^hi^CD11b^+++^F4/80^-^Gr1^+^1A8^+ ^cells are neutrophils. We find that approximately 60% of the CD45^hi ^cell population in the brain at 18 hpi are inflammatory monocytes and approximately 20% are neutrophils (Figure 2G).

**Figure 3:**
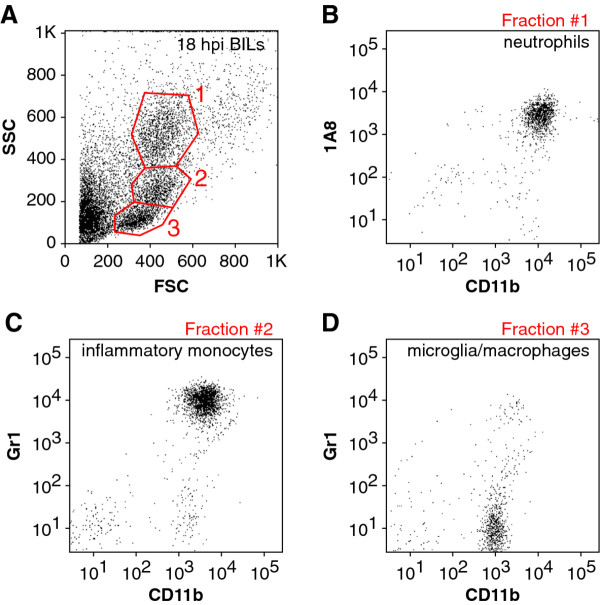
**Flow cytometric scatter characteristics of brain-infiltrating leukocytes (BILs) from acutely infected mice**. BILs collected from Theiler's murine encephalomyelitis virus (TMEV)-infected mice at 18 hpi were gated by forward scatter (FSC) and side scatter (SSC) profiles into three distinct populations (A). Fraction 1 corresponded to the CD45^hi^CD11b^+++^1A8^+ ^neutrophil fraction (B). Fraction 2 corresponded to the CD45^hi^CD11b^++^Gr1^+ ^inflammatory monocyte population (C). Fraction 3 contained only CD45^mid^CD11b^+^Gr1^-^1A8^- ^microglia (resident macrophages) (D). Results are representative of three mice.

In order to corroborate these working definitions, we carefully examined the forward and side scatter profiles of the 18 hpi BILs population from a large number of mice. Gating of the scatter profiles as shown in Figure 3A yielded three distinct cell populations that consistently displayed almost pure populations of neutrophils (fraction 1, Figure 3B), inflammatory monocytes (fraction 2, Figure 3C), or microglia (resident macrophages; CD45^mid^CD11b^+^Gr1^-^1A8^-^) (fraction 3, Figure 3D) using the flow cytometric definitions provided above. Flow sorting to isolate each of these three populations followed by histological examination of the cells further supported our definitions (data not shown) [17,18].

**Figure 4:**
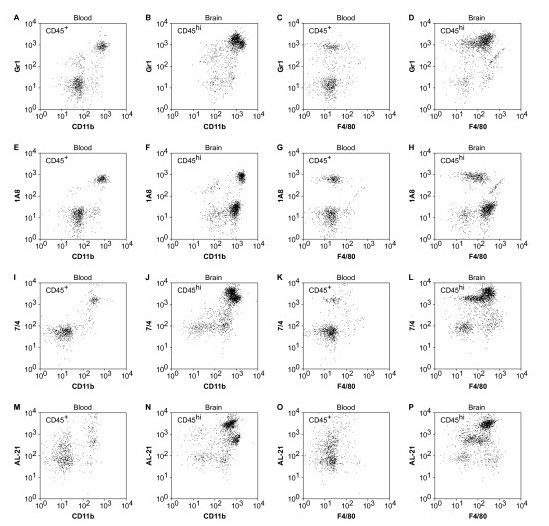
**Characterization of the Ly6 antigen series on brain-infiltrating leukocytes (BILs) and peripheral blood leukocytes (PBLs)**. Flow cytometric analysis of PBLs (A, C, E, G, I, K, M, O) and BILs (B, D, F, H, J, L, N, P) collected from the same mouse 18 h after infection with Theiler's murine encephalomyelitis virus (TMEV) revealed the presence of readily distinguishable neutrophil populations in both cell preparations. However, inflammatory monocytes were only detected in the BILs. Consistent with the analyses performed above, the CD45^hi^CD11b^++^F4/80^+ ^population of inflammatory monocytes was positive for Gr1 (Ly6C/G), 7/4 (Ly6B), and AL-21 (Ly6C) but negative for 1A8 (Ly6G). The CD45^hi^CD11b^+++^F4/80^- ^neutrophil population was positive for all four Ly6 series antigens. Of note, CD45^hi^CD11b^++^F4/80^+ ^inflammatory monocytes in the BILs expressed higher levels of surface Ly6C/G (B, D) and Ly6B (J, L) as compared to CD45^hi^CD11b^+++^F4/80^- ^neutrophils and expressed 10-fold higher levels of the AL-21 antigen (N, P). The CD45^+^CD11b^+ ^macrophage population in the blood was negative for all Ly6 series antigens. Results are representative of five mice.

Confusion exists in the literature regarding the use of Ly6 antigens as markers of neutrophils and inflammatory monocytes. Historically, Gr1 staining of the Ly6C/G heteroantigen was used to define neutrophils. More recently, it has become clear that Gr1 marks both neutrophils and a population of inflammatory monocytes. The availability of the 1A8 marker, which only recognizes Ly6G and only labels neutrophils, now allows for better resolution of these populations [15,19]. In order to further validate our phenotype definitions, we also examined the Ly6 series in blood at 18 hpi and compared the profile to BILs from the same animal (Figure 4). In general, the staining for Gr1 (Ly6C/G) (Figure 4A-D) and 1A8 (Ly6G) (Figure 4E-H) was higher on the brain-infiltrating cells as compared to the same populations in the blood. Of note, brain-infiltrating inflammatory monocytes exhibited elevated Gr1 staining compared to brain-infiltrating neutrophils (Figure 4B). We were unable to identify a population of inflammatory monocytes in the blood in the infected mice, suggesting either that these cells are not circulating at this time or that so many have been recruited to the brain that the blood levels are reduced to below detection (Figure 4A, E, I, M). Surface 1A8 labeling was increased on neutrophils that had entered the brain (Figure 4F) as compared to circulating neutrophils (Figure 4E), suggesting that this molecule may be upregulated as neutrophils home to target tissues. Finally, inflammatory monocytes exhibited higher surface levels of 7/4 (Ly6B-specific) and AL-21 (Ly6C-specific) staining as compared to neutrophils in the brain (Figure 4J, L, N, P). Based on these observations, in addition to differential surface CD11b, Gr1, and 1A8 staining, we can further distinguish brain-infiltrating monocytes from brain-infiltrating neutrophils on the basis of surface expression of the 7/4 and AL-21 antigens. Brain-resident microglia/macrophages were consistently negative for all Ly6 antigens (not shown).

**Figure 5:**
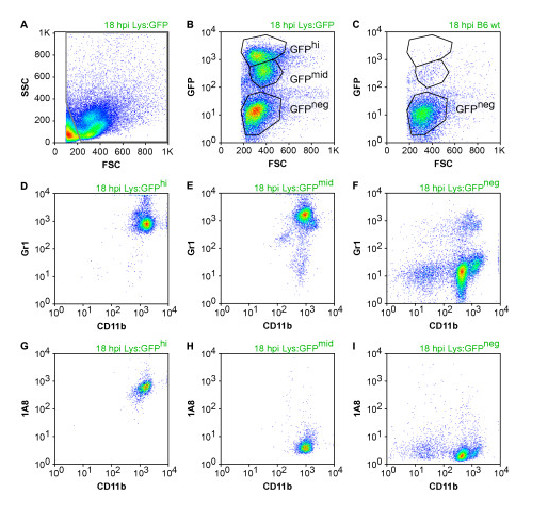
**Assessment of the green fluorescent protein (GFP)^+ ^brain infiltrate in Theiler's murine encephalomyelitis virus (TMEV)-infected LysM:eGFP mice**. Flow cytometric analysis of brain-infiltrating leukocytes (BILs) collected at 18 hpi from LysM:eGFP reporter mice (Lys:GFP) and from wild-type littermates (wt) (C) revealed the presence of distinct GFP^hi ^and GFP^mid ^populations in the reporter mice (B). Further characterization of CD11b, Gr1, and 1A8 staining showed that the GFP^hi ^population was composed of CD11b^+++^Gr1^+^1A8^+ ^neutrophils, the GFP^mid ^population was composed of CD11b^++^Gr1^+^1A8^- ^inflammatory monocytes, and the GFP^neg ^population contained CD11b^+^Gr1^-^1A8^- ^microglia (resident macrophages). (D-I) are derived from the gated populations in (B). These findings confirm our other observations and indicate the presence of both neutrophils and inflammatory monocytes in the brain at 18 hpi. Results are representative of more than 10 mice.

**Figure 6:**
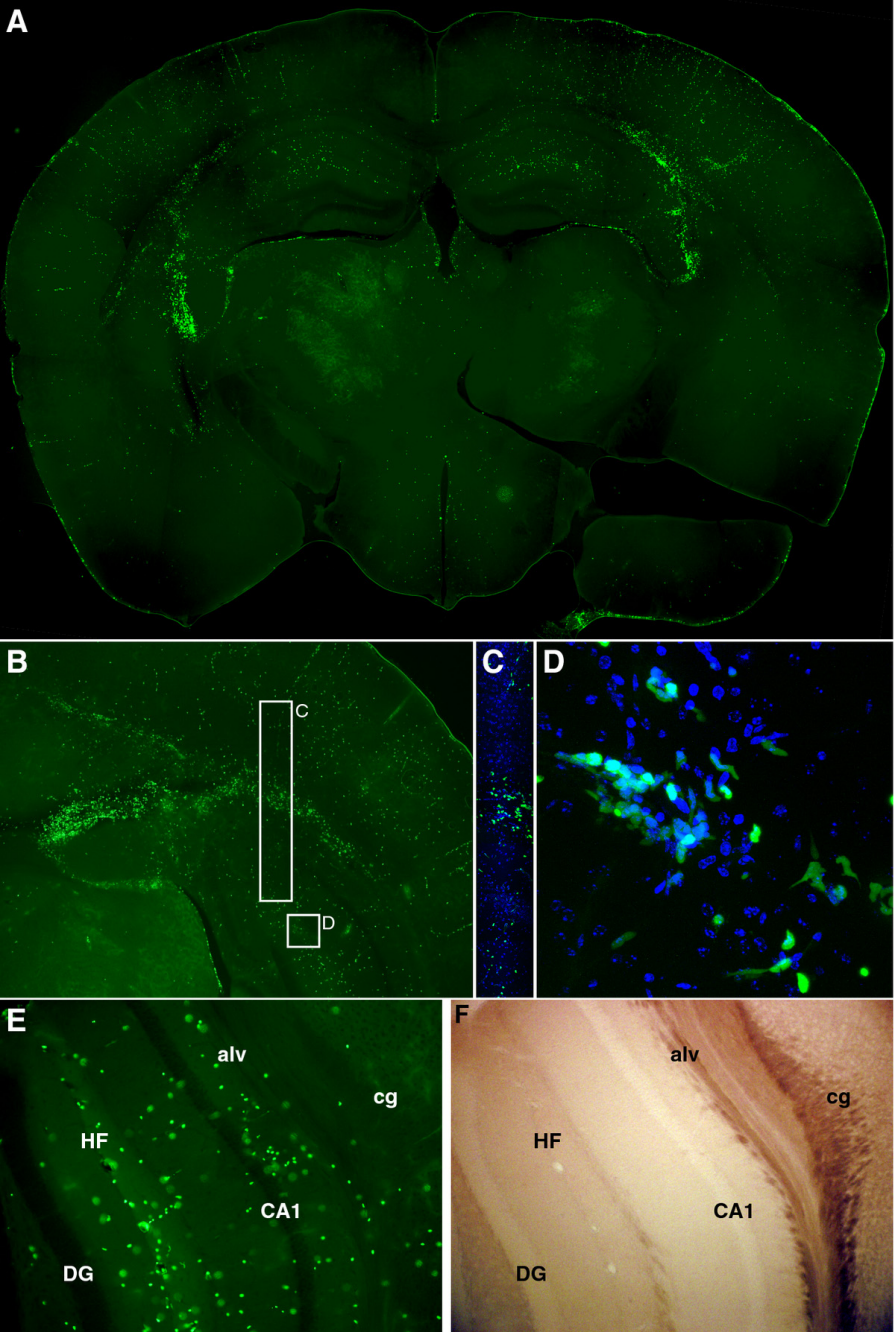
**Visualization of the green flourescent protein (GFP)^+ ^infiltrate in the brain of Theiler's murine encephalomyelitis virus (TMEV)-infected LysM:eGFP mice**. TMEV-infected LysM:eGFP reporter mice were killed by intracardiac perfusion of 4% paraformaldehyde 18 h after infection. Vibratome sections were prepared and analyzed by epifluorescence microscopy. The distribution of GFP^+ ^neutrophils and inflammatory monocytes at low magnification (A, B) matches the distribution of inflammatory infiltrate observed in Figure 1. Higher magnification of a cross-section through the alveus and corpus callosum (C) and at the hippocampal fissure (D) shows the presence of both GFP^hi ^and GFP^mid ^cells with a variety of morphologies consistent with migratory behavior. Comparison of the epifluorescent signal (E) and the brightfield image (F) of a cross-section of the hippocampus confirms the presence of numerous GFP^+ ^neutrophils and inflammatory monocytes at the hippocampal fissure (HF), in the alveus (alv), and proximal to the pyramidal neurons of CA1. Cg = cingulum bundle; DG = dentate gyrus. Green represents GFP in (A-E), blue in (C-D) shows 4',6-diamidino-2-phenylindole (DAPI). Results are representative of eight mice.

We further confirmed the presence of neutrophils and inflammatory monocytes in the brain following infection using LysM:GFP reporter mice. These animals express enhanced green fluorescent protein (GFP) at the lysozyme M locus, yielding GFP^hi ^neutrophils and GFP^mid ^monocytes [10]. No other cells express GFP in these mice, making them an excellent tool for tracking neutrophils and inflammatory monocytes. We observed robust and clearly distinguished populations of GFP^hi ^and GFP^mid ^cells in the BILs at 18 hpi (Figure 5B). Gating into GFP^hi^, GFP^mid^, and GFP^neg ^populations confirmed that our CD11b^+++^Gr1^+^1A8^+ ^neutrophil population was almost exclusively GFP^hi ^(Figure 5D, G), while our CD11b^++^Gr1^+^1A8^- ^inflammatory monocyte population was almost exclusively GFP^mid ^(Figure 5E, H). The GFP^neg ^population in the 18 hpi BILs exhibited a CD11b^+^Gr1^-^1A8^- ^immunophenotype consistent with microglia (resident macrophages) (Figure 5F, I). The clear distinction between GFP^+ ^and GFP^- ^cells in the BILs population was further exploited by assessing the physical location of infiltrating GFP^+ ^cells in vibratome sections of brain from 18 hpi mice (Figure 6). We found that the majority of GFP^+ ^cells were located in proximity to the hippocampal formation (Figure 6A), with high concentrations of labeled cells just superior and lateral to the hippocampus proper. Higher magnification also showed dense clusters of GFP^+ ^cells in the corpus callosum and alveus (Figure 6B, C, E) and in the hippocampal fissure (Figure 6D, E). This suggests that the white matter tracts overlying the hippocampus and the vasculature-rich hippocampal fissure are primary sites for infiltration of neutrophils and inflammatory monocytes. These observations are consistent with the histological analyses shown in Figure 1 and further support a model in which neutrophils and inflammatory monocytes are rapid responders to brain infection. Of note, the robust localization of these cells to the hippocampal formation is consistent with our previously published observations that the hippocampus is the primary site of pathology following TMEV infection and that this damage is not a direct result of the virus but is rather a bystander phenomenon [8].

### Neutrophils and inflammatory monocytes enter the brain within 12 h of infection

**Figure 7:**
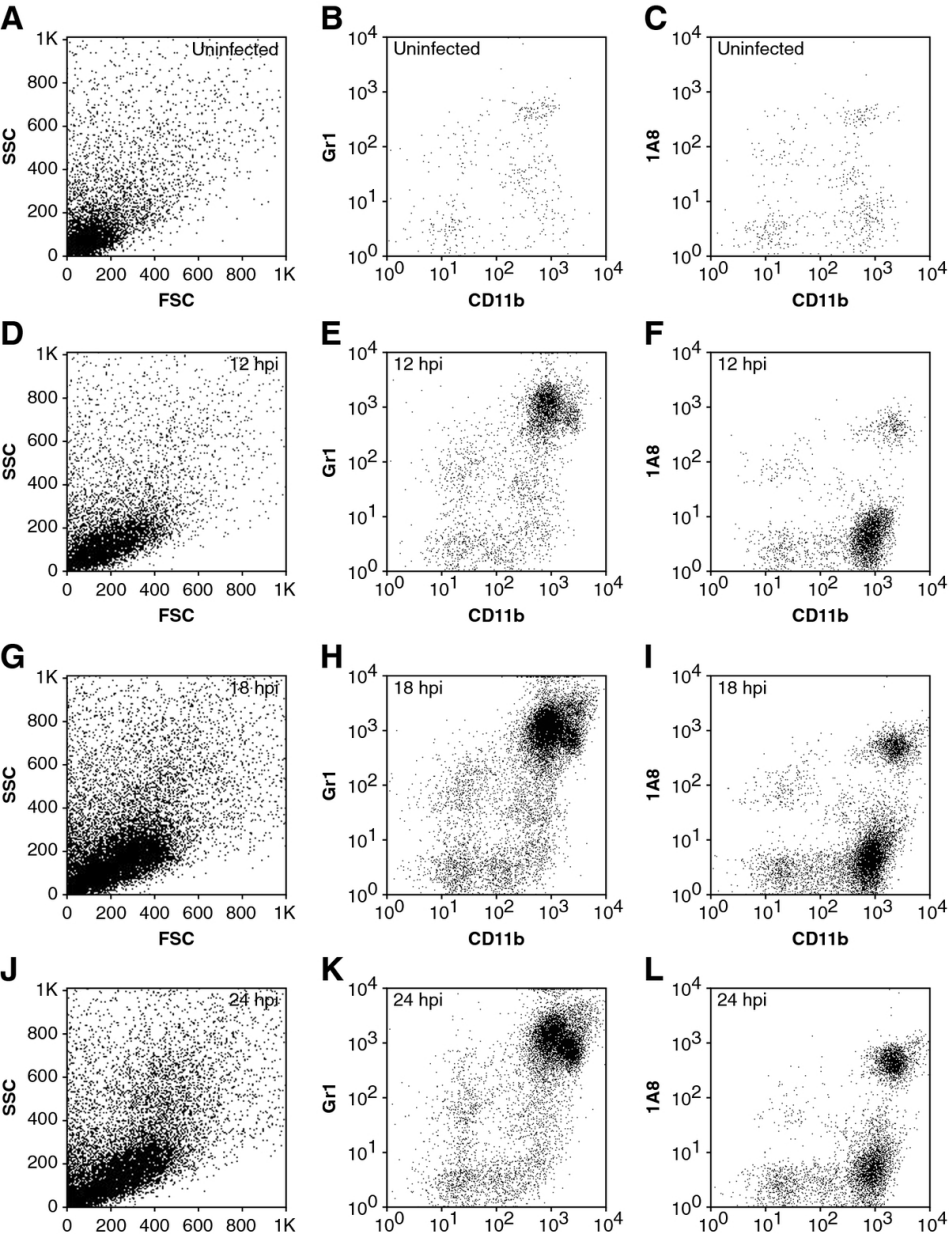
**Flow cytometric assessment of the timecourse of neutrophil and inflammatory monocyte infiltration during acute infection**. Brain-infiltrating leukocytes (BILs) were prepared from uninfected (A-C) mice or from mice infected with Theiler's murine encephalomyelitis virus (TMEV) for 12 h (D-F), 18 h (G-I), or 24 h (J-L). Cells were stained with antibodies against CD45, CD11b, Gr1 (Ly6C/G) and 1A8 (Ly6G) and analyzed by flow cytometry. Essentially no Gr1^+ ^(B) or 1A8^+ ^cells (C) were observed in uninfected mice and the forward scatter (FSC) vs side scatter (SSC) plot showed a clear absence of the larger, more granular cells present at later time points (A). In contrast, TMEV-infected mice exhibited both CD11b^++^Gr1^+ ^inflammatory monocytes and CD11b^+++^1A8^+ ^neutrophils as early as 12 hpi (D-E), with inflammatory monocytes outnumbering neutrophils (E, F). Both of these populations increased in number by 18 hpi (G-I). Neutrophils continued to increase in the infiltrate by 24 hpi (K, L) while inflammatory monocytes began to decrease at this time point (K, L). The granular profile of neutrophils was also clearly present by FSC and SSC at 24 hpi (J). Results are representative of more than 20 mice at each timepoint.

BILs were prepared from uninfected mice and at 12, 18, and 24 hpi (Figure 7) in order to determine how rapidly neutrophils and inflammatory monocytes enter the brain. We found a robust population of inflammatory monocytes present at 12 hpi (Figure 7E, F), suggesting that the cells had started infiltrating even earlier. Analysis of BILs at 6 hpi showed a small but inconsistent population of inflammatory monocytes (data not shown), indicating that infiltration began in earnest between 6 and 12 hpi. A small population of neutrophils was present in the BILs by 12 hpi (Figure 7E, F). By 18 hpi the density of both neutrophils and inflammatory monocytes had increased (Figure 7H, I). By 24 hpi the number of neutrophils continued to increase while the number of inflammatory monocytes decreased relative to 18 hpi (Figure 7K, L). Cell counts and relative percent of each cell population are provided in Table 1.

**Table 1:** Quantitation of brain infiltrating inflammatory monocytes and neutrophils (brain-infiltrating leukocytes (BILs)) at 12, 18, and 24 h post infection (hpi)

Timepoint	Number of inflammatory monocytes	Number of neutrophils	Inflammatory monocytes as percentage of CD45^hi ^BILs	Neutrophils as percentage of CD45^hi ^BILs
0 hpi	17 ± 11	3 ± 3	-	-

12 hpi	1,151 ± 270	106 ± 40	60.6 ± 28.9	5.7 ± 3.5

18 hpi	4,299 ± 381	1,228 ± 127	57.5 ± 8.9	16.4 ± 2.8

24 hpi	3,226 ± 250	1,645 ± 231	51.5 ± 7.7	26.3 ± 5.6

**Figure 8:**
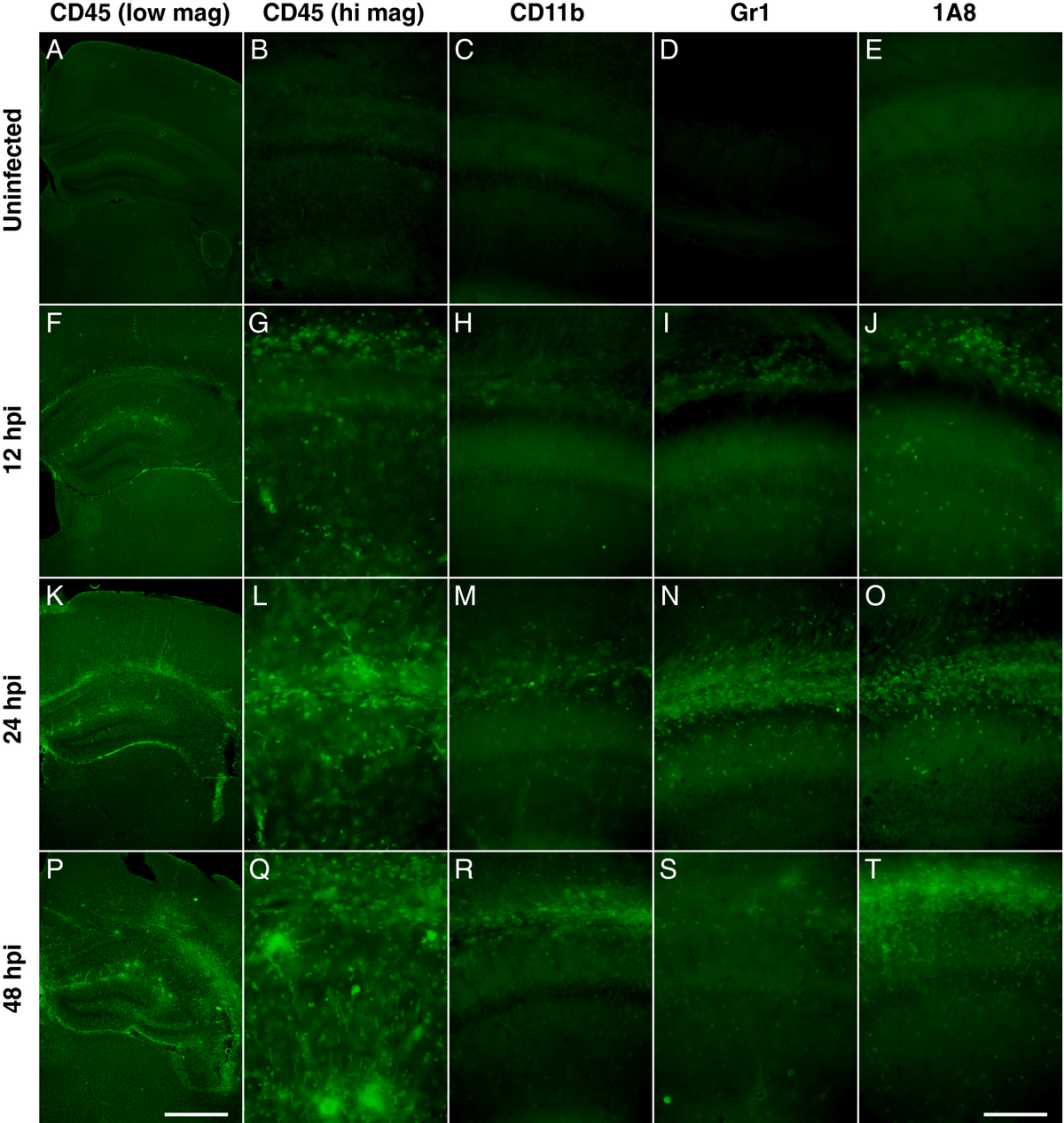
**Visualization of the timecourse of neutrophil and inflammatory monocyte infiltration during acute infection**. Uninfected mice (A-E) or mice infected with Theiler's murine encephalomyelitis virus (TMEV) for 12 h (F-J), 24 h (K-O), or 48 h (P-T) were killed by intracardiac perfusion of 4% paraformaldehyde and vibratome sections of brain were prepared and stained with CD45 (A, B, F, G, K, L, P, Q), CD11b (C, H, M, R), Gr1 (D, I, N, S), or 1A8 (E, J, O, T). No staining for any of the markers was observed in the uninfected brain (A-E). The number of CD45-labeled cells increased within the hippocampal region through 12, 24, and 48 hpi (F, K, P). Higher magnification of a cross section showing the corpus callosum and alveus (top of each image), the CA1 pyramidal neuron layer (middle of each image), and the hippocampal fissure (bottom of each image) (B-E, G-J, L-O, Q-T) confirms the increasing density of the CD45^+ ^infiltrate and also reveals that the Gr1^+ ^population peaks at 24 h (N) while the 1A8^+ ^population continues to increase through time (J, O, T). The scale bar in P is 1 mm and refers to (A), (F), and (K). The scale bar in T is 200 μm and refers to the remaining panels. Results are representative of more than five mice at each timepoint.

Immunostaining brain sections at these time points revealed a growing population of CD45^+ ^cells in the hippocampal fissure and in the superior white matter tracts (Figure 8A, B, F, G, K, L, P, Q). Many of these cells were CD11b^+ ^(Figure 8C, H, M, R) and a strong population of Gr1^+ ^cells was observed at 24 hpi (Figure 8N) but was greatly decreased by 48 hpi (Figure 8S). Likewise, 1A8^+ ^cells were clearly present in the hippocampus at 12, 24, and 48 hpi (Figure 8J, O, T). The relative differences in number of cells labeled with each of these markers is hard to interpret, especially given the overall difficulty in successfully immunostaining for several of the markers. Nonetheless, Figure 8 indicates that the hippocampus is a target for the rapid infiltration of CD45^+^CD11b^+^Gr1^+ ^and CD45^+^CD11b^+^1A8^+ ^cells, confirming the findings at 18 hpi in the LysM:GFP mice (Figure 5, Figure 6) and providing anatomic localization to the observations in Figure 7.

### Inflammatory monocytes injure the hippocampus

**Figure 9:**
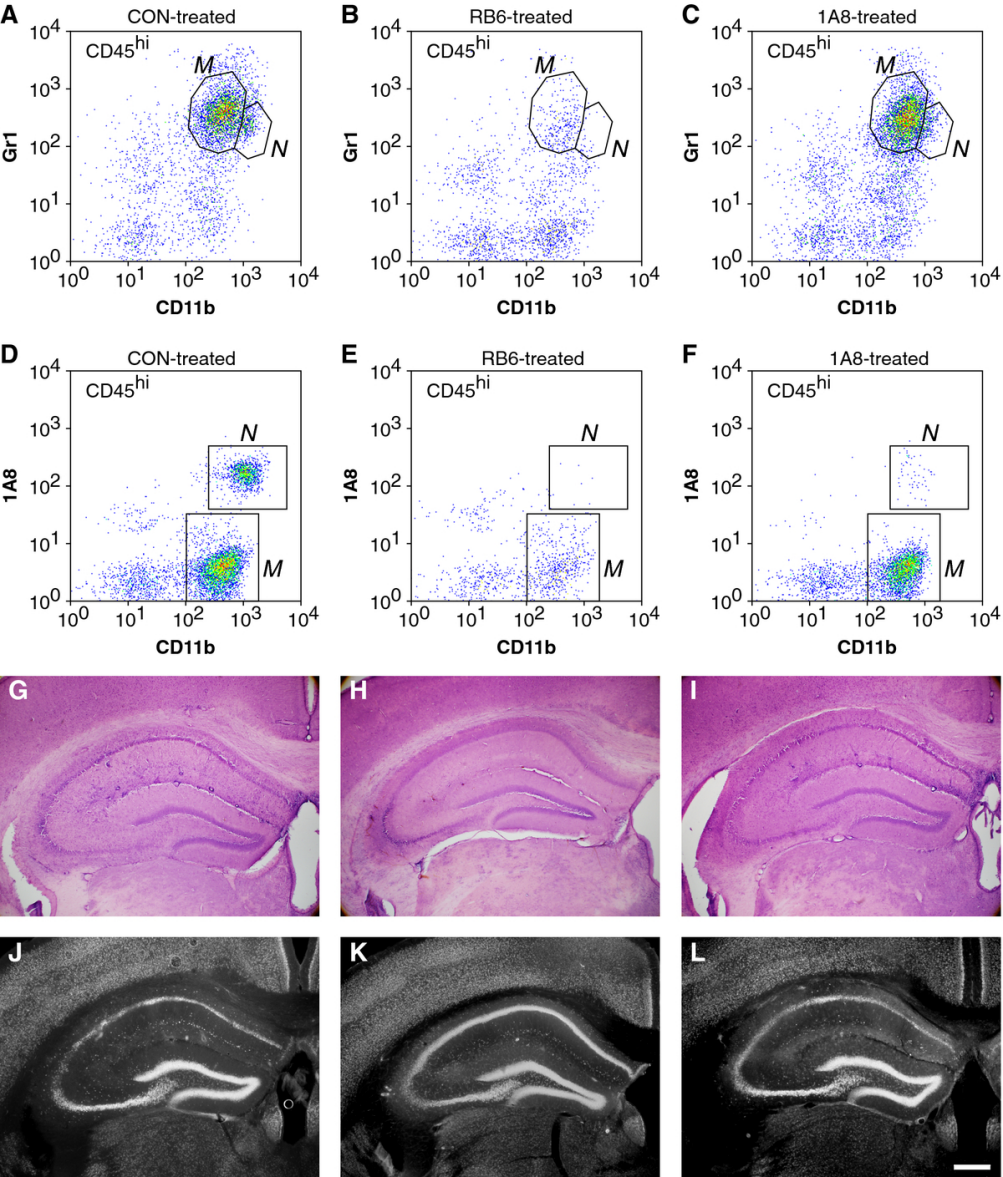
**Immunodepletion of inflammatory monocytes but not neutrophils protects the hippocampus from injury**. Theiler's murine encephalomyelitis virus (TMEV)-infected mice were treated with RB6-8C5 to deplete inflammatory monocytes and neutrophils (B, E, H, K), 1A8 to deplete only neutrophils (C, F, I, L), or were treated with vehicle (A, D, G, J). Brain-infiltrating leukocytes (BILs) were collected at 18 hpi and inflammatory monocytes and neutrophils were counted by flow cytometry (A-F). RB6 treatment abrogated most of the inflammatory monocyte (B) and neutrophil response (E). 1A8 treatment abrogated the neutrophil response (F) but not the inflammatory monocyte response (C). Histological analysis of hippocampal injury (G-I) and staining for the neuron-specific marker NeuN (J-L) revealed that depletion of inflammatory monocytes and neutrophils protected the hippocampus (H) and preserved the neurons in CA1 (K) whereas depletion of only neutrophils had no effect on the injury (I, L). Scale bar in L is 500 μm and refers to G-L. *M *= inflammatory monocyte; *N *= neutrophil. The flow cytometry results are representative of 10 mice per group. The histological analyses are representative of at least five mice per group.

**Figure 10:**
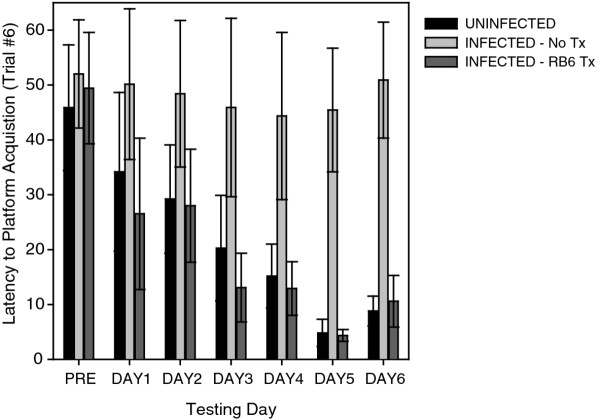
**Immunodepletion of inflammatory monocytes preserves cognitive function in the Morris water maze**. Uninfected mice, untreated Theiler's murine encephalomyelitis virus (TMEV)-infected mice (No Tx), and infected mice treated with RB6-8C5 to deplete inflammatory monocytes and neutrophils (RB6 Tx) were assessed in the Morris water maze starting at 14 dpi. Following 3 days of training on a visible platform, latency to hidden platform acquisition was measured over six trials each day for 6 days. Pretesting latency to the hidden platform is shown (PRE) and compared to the latency to acquisition during the sixth and final trial of each day (DAY1 to DAY6). The uninfected mice readily learned the maze and navigated to the escape platform in less than 10 s by day 5. RB6-8C5 immunodepleted mice performed as well as uninfected controls. In contrast, the infected mice that were not depleted were unable to learn the maze at any point during the testing phase. These observations are consistent with the hippocampal pathology findings in Figure 9. Results are from 10 mice in each group.

We have previously hypothesized that the innate immune response to acute TMEV infection mediates subsequent hippocampal injury that leads to cognitive deficits [8]. In order to distinguish the role of inflammatory monocytes from neutrophils in this injury, we immunodepleted these populations and assessed hippocampal pathology (Figure 9) and cognitive performance (Figure 10). Neutrophils and inflammatory monocytes were simultaneously depleted by daily treatment from -2 dpi to +2 dpi with intraperitoneal injection of purified RB6-8C5 (500 μg/day; rat anti-Gr1). Neutrophils but not inflammatory monocytes were depleted by daily injection from -2 dpi to +2 dpi of purified 1A8 (500 μg/day; mouse anti-Ly6G). Controls received daily injections of PBS. BILs prepared at 18 hpi from control mice showed 4,898 ± 191 inflammatory monocytes and 1,515 ± 192 neutrophils (Figure 9A, D); 22.9% ± 1.0% of the BILs were CD45^hi^. Control mice showed robust hippocampal pathology at 7 dpi as assessed by hematoxylin and eosin (H&E) histology (Figure 9G) and immunostaining for the neuronal marker NeuN (Figure 9J). Regions of no or weak NeuN staining in the CA1 layer in Figure 9J represent neuronal dropout or pyknotic neurons. In contrast, RB6-treated mice had only 106 ± 34 inflammatory monocytes and 11 ± 3 neutrophils in the 18 hpi BILs (Figure 9B, E) and only 6.7% ± 0.4% of the BILs were CD45^hi^. Moreover, the hippocampus was completely preserved in these mice at 7 dpi (Figure 9H, K). The thick, multi-cell-layered NeuN staining in the CA1 layer in Figure 9K is indistinguishable from uninfected mice (data not shown). The reduction in inflammatory monocytes and neutrophils was highly significant versus control BILs (F(2,28) = 1,141.175, *P *< 0.001; inflammatory monocytes in control vs RB6: q(28,2) = 63.454, *P *< 0.001; neutrophils in control vs RB6: q(28,3) = 19.923, *P *< 0.001). However, while treatment with 1A8 almost completely abrogated the neutrophil response in the 18 hpi BILs (66 ± 24 neutrophils; control vs 1A8: q(28,2) = 17.917, *P *< 0.001) (Figure 9F) there was an actual increase in the number of inflammatory monocytes (6,251 ± 237 inflammatory monocytes; control vs 1A8: q(28,2) = 19.194, *P *< 0.001) (Figure 9C). The percentage of CD45^hi ^cells in the BILs following 1A8 treatment was 23.4% ± 1.3% (control vs 1A8: q(28,2) = 1.064, *P *= 0.458; RB6 vs 1A8: q(28,3) = 34.188, *P *< 0.001). Critically, the hippocampus was robustly injured in the 1A8-treated mice (Figure 9I, L). Finally, the absence of hippocampal injury in the RB6-treated mice led to the preservation of cognitive function as assessed by Morris water maze (Figure 10). Infected, RB6-treated mice showed the same ability to learn in the maze as uninfected mice (F(2,28) = 15.508, *P *< 0.001; uninfected vs RB6-treated: q(28,2) = 1.089, *P *= 0.441) while infected, control-treated mice were completely unable to learn to navigate the maze (uninfected vs infected, control-treated: q(28,2) = 14.826, *P *< 0.001; RB6-treated vs control-treated: q(28,3) = 15.519, *P *< 0.001) [1,8]. We interpret these findings as strong support for our hypothesis that neutrophils and inflammatory monocytes are first responders to TMEV infection in the brain and that inflammatory monocytes are primarily responsible for hippocampal damage and loss of cognitive function.

## Conclusions

In contrast to other viral models in which neutrophils apparently do not infiltrate the CNS until recruited by other immune populations such as lymphocytes [2], our findings suggest that neutrophils are one of the earliest responders in the TMEV model. As in other viral model systems [4-6], we predict that these cells will serve to prime the way for the adaptive response. In addition, our time course findings suggest that inflammatory monocytes precede neutrophils into the brain and the 1A8 depletion experiments show that inflammatory monocytes are competent to enter the CNS in the absence of neutrophils. Indeed, in the absence of neutrophils there was a significant increase in the number of inflammatory monocytes in the brain at 18 hpi, suggesting that neutrophils may exert a regulatory effect that slows or reverses inflammatory monocyte accumulation in the brain. The depletion experiments also show that inflammatory monocytes are required for the loss of CA1 pyramidal neurons that occurs in the first few days of infection [8] while neutrophils appear to be dispensable for this injury and the downstream consequences. Ongoing experiments will assess the impact of inflammatory monocytes versus neutrophils in the recruitment of the adaptive immune system and eventual control of the virus. Likewise, ongoing experiments in chemokine receptor knockout hosts will determine the relative impact of different chemotactic pathways in the separate and integrated neutrophil and inflammatory monocyte responses.

Our findings indicate that neutrophils and inflammatory monocytes rapidly and robustly respond to TMEV infection by infiltrating the brain. We hypothesize that these effector cells, and inflammatory monocytes in particular, may be important therapeutic targets for immunomodulatory or immunosuppressive therapies aimed at reducing or preventing CNS pathology associated with acute viral infection.

## Competing interests

The authors declare that they have no competing interests.

## Authors' contributions

CLH designed all experiments, analyzed all data, and executed all of the microscopy. RLC prepared all of the animals and executed all of the experiments except immunostaining and MWM. RSS sectioned the tissue and performed the immunostaining. SJL performed the Morris water maze experiments. All authors read and approved the final version of the manuscript.
